# Comparative analysis of mitochondrial genomes in genetically distinct groups of the dojo loach *Misgurnus anguillicaudatus*

**DOI:** 10.1080/23802359.2020.1840937

**Published:** 2020-12-24

**Authors:** Kiko Shibata, Duong Thuy Yen, Takafumi Fujimoto, Katsutoshi Arai

**Affiliations:** a Graduate School of Fisheries Sciences, Hokkaido University, Hakodate, Japan; b College of Aquaculture and Fisheries, Can Tho University, Can Tho, Vietnam; c Faculty of Fisheries Sciences, Hokkaido University, Hakodate, Japan; d Institute for the Advancement of Higher Education, Hokkaido University, Sapporo, Japan

**Keywords:** Dojo loach, *Misgurnus anguillicaudatus*, mitochondrial genome

## Abstract

Dojo loach (*Misgurnus anguillicaudatus*) that inhabit Japan are composed of two genetically divergent groups (A and B). Although most individual loach reproduce bisexually, clone lineages exist in certain populations that reproduce gynogenetically. To investigate the molecular phylogenetic relationships among the *M. anguillicaudatus* groups and clone lineages, complete mitogenomes of members from groups A and B and a clone lineage were sequenced using long range PCR and primer walking methods. The three groups of mitogenomes shared the same gene order and had similar base compositions and codon usage patterns. Phylogenetic analysis indicated group A and the clone lineage were genetically close with group B being genetically divergent.

Dojo loach (*Misgurnus anguillicaudatus*) are small freshwater fish widely distributed along the eastern coasts of Asia from the Amur River to the northern regions of Vietnam (Global Biodiversity Information Facility, 2020). Previous studies using the mitochondrial DNA control region (mtDNA-CR) and two nuclear genes, *recombination activating gene 1* (*RAG1*) and *interphotoreceptor retinoid-binding protein 2* (*IRBP2*) revealed *M. anguillicaudatus* in Japan can be genetically discriminated into three groups, A, B1, and B2 (Morishima et al. 2008, Yamada et al. 2015). Although most *M. anguillicaudatus* in nature commonly reproduces bisexually, gynogenetically reproducing clone lineages are distributed in certain districts in Japan (Morishima et al. 2002). Based on phylogenetic and cytogenetic studies, the clone lineages are thought to be hybrids originating between group A females and group B1 males (Yamada et al. 2015, Kuroda et al. 2018).

Complete mitogenomes of *M. anguillicaudatus*, independent of nuclear genomes, have been reported in previous studies (He et al 2008, Zhang et al 2018, Zhang et al 2019). Here, we analyzed the mitogenomes of group A, group B1, and a clone lineage of *M. anguillicaudatus* and report their molecular phylogenetic relationship among the *Misgurnus* genus. Specimens of *M. anguillicaudatus* group A, group B1, and a clone lineage were collected from Hokkaido prefecture. Specifically, the group A and clone lineage specimens were collected from Ozora, Japan and the group B1 specimen were collected from Nanae, Japan. Genotypes of the nuclear genomes were previously confirmed by Fujimoto et al. (2017). All specimens have been deposited in the DNA collection of the Hokkaido University Museum, Hakodate, Japan (catalog number: HUMZ 231155–231157).

Mitogenomes of the three groups were sequenced using long range PCR and primer walking methods (Miya and Nishida 1999). The complete genomes were 16,566 bp for group A (accession number LC532166), 16,641 bp for group B1 (accession number LC532167), and 16,568 bp for the clone lineage (accession number LC532168). The mitogenomes of all three groups included 13 protein-coding genes (PCGs), two ribosomal RNA (rRNA) genes, 22 transfer RNA (tRNA) genes, one control region, and the origin of light strand replication (OriL), consistent with what is found in other teleost fish. The NADH-ubiquinone oxidoreductase chain 6 protein gene (*ND6*) and eight of the tRNA genes were encoded on the L-strand with the other genes encoded on the H-strand. The group A and clone lineage specimens shared the same gene directions and sequence lengths. In comparison, these genes in group B1 also had the same direction, but two PCGs (*COII* and *ND3*) and two rRNA genes had sequence lengths that differed from those of group A and the clone lineage. The initiation codons of the PCGs, order of the tRNAs, and location of the two of the rRNA genes were consistent with mitogenomes of other *M. anguillicaudatus* isolates. Based on pairwise comparisons, mitogenome sequence similarity was 86.22% between groups A and B1, 95.71% between group A and the clone lineage, and 86.17% between group B1 and the clone lineage.This was consistent with the findings of Morishima et al. (2008), who showed that the clone lineage had inherited their mitochondria from group A strains.

A phylogenetic relationship of *Misgurnus* spp. was reconstructed based on their mitogenomes using CLC Genomics Workbench (ver. 9.5.3) and the neighbor-joining method. The resulting phylogenetic tree consisted of three major clades (Figure 1). The first clade contained only *M. anguillicaudatus* strains for which the sequences were previously deposited by Zeng et al. (2012), Yu et al. (2014a, 2014b, 2014c), Zhou et al. (2014), Lee et al. (2018), Zhang et al. (2018), and Zhang et al. (2019). The second clade contained a *M. anguillicaudatus* isolate and another isolate of group B1, as well as two *M. bipartitus* isolates. The sequences for these isolates were previously deposited by Huang et al. (2015) and Miya et al. (2015). The third clade contained *M. anguillicaudatus* isolates, another isolate of group A, and the clone lineage isolated from the current study, in addition to other *Misgurnus* species including *M. mizolepis*, *M. mohoity*, and *M. nikolskyi* for which the sequences were previously deposited by He et al. (2008) and Saitoh et al. (2006). The results support the previous report that *M. anguillicaudatus* consists of genetically diverse groups (Zhang et al. 2018). The results also suggest that group B1 *M. anguillicaudatus* strains, which are distributed mainly in Japan, are closely related to *M. bipartitus*. Meanwhile, group A and the clone lineage were genetically closer related to *M. mohoity* and *M. nikolskyi* than to group B1. Further research regarding the mitogenome and nuclear genome with respect to geographical and morphological data will help solve the taxonomical complexity of the genus *Misgurnus*.

**Figure 1.**
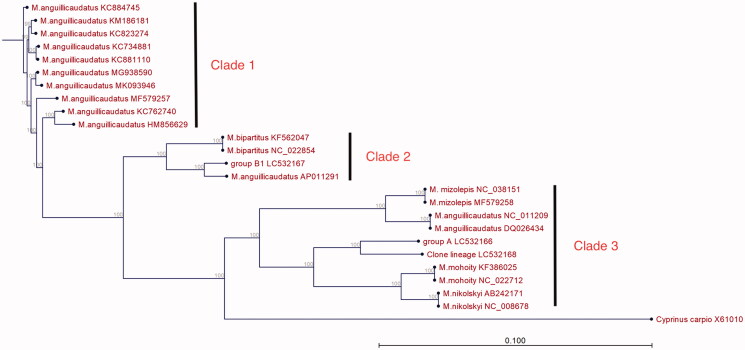
Phylogenetic relationship of *Misgurnus* spp. based on their mitogenomes. The phylogenetic tree was constructed using CLC Genomics Workbench (ver. 9.5.3) and the neighbor-joining method. Numbers above branches indicate bootstrap values for 1000 replicates. The phylogenetic tree consists of three major clades.

## Data Availability

The data that support the findings of this study are available in ‘DNA Date Bank of Japan’ at http://getentry.ddbj.nig.ac.jp/, accession number [LC532166, LC532167, LC532168].
